# Validation of the internet addiction test (IAT) to Sinhalese and assessment of internet addiction among school children in Sri Lanka

**DOI:** 10.1192/bjo.2021.127

**Published:** 2021-06-18

**Authors:** Kavinda Gunathillaka, Chamara Wijesinghe, Trivon Gunasekera, Bhagye Premathilake, Asiri Rodrigo, Arunasalam Pathmeswaran, Lalith Kuruppuarachchi

**Affiliations:** 1 Department of Psychiatry, Faculty of Medicine, University of Kelaniya; 2 University Psychiatry Unit, Colombo North Teaching Hopsital; 3 Department of Public Health, Faculty of Medicine, University of Kelaniya

## Abstract

**Aims:**

To translate Young's Internet Addiction Test (IAT) to Sinhalese and validate for use in a Sri Lankan population. Following validation of the questionnaire, to use the validated questionnaire to assess the prevalence of internet addiction in a school going population in the Western province of Sri Lanka and identify characteristics of those addicted to the internet.

**Background:**

The internet is widely used across the world and in Sri Lanka. Though essential for everyday life there are many negative aspects of internet use. Addiction to the internet is one such problem and identified to exist among the general population and students in other countries. The most common tool used to measure internet addiction is Young's internet addiction test. The phenomena of internet addiction has not been scientifically studied in Sri Lanka according to our knowledge.

**Method:**

A school-based cross-sectional analytical study conducted in two stages among students aged 15 to 19 years. In stage 1 of the study, 200 students were administered the Sinhalese translation of the IAT and internal consistency and test retest validity assessed. Once validation of the scale was established the translated scale was used on a sample of 2800 students to assess presence of internet addiction.

**Result:**

The Sinhala translation of the Internet addiction test showed good reliability and validity The Chrohnbach's alpha value was 0.78 and Pearson correlation coefficient of 0.85 and therefore suitable to use in a Sinhalese speaking population in Sri Lanka.

Internet addiction was identified among the study population. 8% of the entire study sample and 12.6% among those using the internet showed features of internet addiction. The majority of cases of internet addiction identified were mild 8.2% followed by moderate internet addiction in 3.6% and only 0.9 % having severe internet addiction. There were no significant demographic or internet use related features identified among those with internet users and those not addicted to the internet.

**Conclusion:**

This study demonstrated that the Sinhala translation of Young's IAT is suitable to assess internet addiction in Sri Lanka. It also identified that there are students in Sri Lanka who are addicted to the internet. This will possibly impact negatively on their lives at a crucial stage of development and have immediate as well as long term detrimental effects. More studies are required to identify characteristics of those who are addicted to the internet and to plan interventions.

